# Demographic and motivational differences between participants in analog and digital citizen science projects for monitoring mosquitoes

**DOI:** 10.1038/s41598-023-38656-y

**Published:** 2023-07-31

**Authors:** Berj Dekramanjian, Frederic Bartumeus, Helge Kampen, John R. B. Palmer, Doreen Werner, Nadja Pernat

**Affiliations:** 1 Department of Political and Social Sciences, Universitat Pompeu Fabra, Barcelona, Spain; 2 Centre d’Estudis Avançats de Blanes (CEAB-CSIC), Blanes, Spain; 3 Friedrich-Loeffler-Institut, Greifswald, Insel Riems Germany; 4 Leibniz-Centre for Agricultural Landscape Research (ZALF), Müncheberg, Germany; 5 Institute of Landscape Ecology, University of Münster, Münster, Germany; 6 Centre de Recerca Ecològica i Aplicaicons Forestals (CREAF), Barcelona, Spain; 7 Institució Catalana de Recerca i Estudis Avançats (ICREA), Barcelona, Spain

**Keywords:** Human behaviour, Climate change, Public health, Biodiversity, Invasive species, Urban ecology

## Abstract

Worldwide, citizen scientists help to map the distribution of native and introduced mosquito species in a variety of programs, contributing to environmental research and management decisions. Participant background, behavior, and engagement may vary depending on the project design, especially between those using digital apps and those using physical samples, which in turn may impact the quality and representativeness of the data collected. During the analysis of the responses to a survey directed at citizen scientists participating in  a digital and an analog program, we found significant differences in the respondents’ demographic backgrounds. Diverse participant motivations and varying sentiments towards mosquitoes were observed, indicating differing susceptibility in response to the program messages. The results can be translated into recommendations to further strengthen the appeal of citizen science projects and to promote and sustain public engagement in environmental research.

## Introduction

In recent years, the number and volume of publications using citizen science as the source of research data have increased, and corresponding projects involving the public within various scientific fields have seen a growing number of participants. Characterized as the participation of non-professional scientists in professional science projects^1^, citizen science has grown in relevance, as the past decade has seen a massive growth in citizen science activities and participant volunteers^2,3^. Accordingly, the variety of projects along a gradient from structured over semi-structured to unstructured approaches is large. At one end of the spectrum, there are detailed and highly organized research projects with well-defined protocols, involving a select group of highly trained participants. On the other end, there are global formats that include simple tasks such as opportunistic recording and data collection. Projects related to ecology, biodiversity, and public health have benefited from the involvement of citizen science, as it allows for the enrolment of a large number of volunteers who can generate data at scales that individual researchers would be unable to achieve. Digitization has boosted programs as new possibilities of designing and managing projects were facilitated by developments in communication technology (e.g., through internet platforms or online marketing campaigns)^4–6^. Social media and email communication have allowed researchers to reach and recruit from a wide audience, while applications on smartphones have enabled participants to collect data with their recording, tagging, and tracking capabilities. Although citizen science projects in ecology remain diverse, recent trends indicate a shift towards mass participation models and simpler approaches, with an increasing number of projects relying solely on computer-based participation^7^. Global, non-taxon-specific observation platforms like *iNaturalist* and *observation.org* gather millions of species records contributing to more than 50% of the total Global Biodiversity Information Facility data^8^. Taxon-specific platforms such as *eBird*, which also has millions of observations, are pioneers in ensuring the quality of opportunistically collected big data^9^.

Scientists in the field have studied multiple facets of involvement in citizen science, not only to better understand the expansion^7^, but also to learn more about the relationship between participant demographics, motivation, and project success in achieving scientific and societal goals. Case studies that have explored the demographic background of participants in specific projects have found that most participants were middle-aged and from high socio-economic status groups^10^. For instance, West et al.^11^ surveyed environmental citizen scientists and detected a correlation between demographic characteristics and variations in the motivation of participants, whereas Parrish et al.^19^ found a correlation between participant age and better task performance as well as project retention. A clearer understanding of the demographics of participation could potentially improve data quality^12–14^ and reduce unequal representation^15^. By doing so, it can address the issue of limited perspectives in acquiring knowledge, which may hamper our overall understanding of the world^16^. From a project management perspective, unequal representation can also endanger project success. The inability to motivate younger citizen scientists in, for example, biodiversity monitoring projects, could jeopardize long-term project continuity^17^.

As the success of projects has been reliant on the recruitment and retention of a diverse set of volunteers, understanding the motivational goals that drive citizen scientists to participate has become increasingly crucial to keeping projects up and running. An extensive list of studies has attempted to elucidate these factors^11,18–21^. To date, various catalysts of participation have been revealed, including the opportunity to spend time in nature^17^ and a primary concern for the environment^22^. Intrinsic motivational factors that drive individuals to initially and continually participate in these projects, such as participant desire to contribute to scientific research^22^ along with background interest in science, have been studied^23^ and contrasted against extrinsic motivations, such as game-based rewards and monetary compensation^24^. Research on motivations for participation and retention in citizen science projects has also shed light on some distinctions between field-based and online projects. In-person social interaction, natural resource conservation, and general interest in conservation have been identified as common motivators for participation in community-based citizen science projects^25–27^. Conversely, the importance of contributing to scientific research and enhancing participants’ online reputations has been found to be emblematic of online participation^28–30^. Additionally, motivations have been shown to vary throughout the duration of an online project^31^ and to be dependent on the quality and quantity of participation in online settings^28^.

However, most of these studies present cases focused on singular projects or broad nationwide reviews, and studies that examine purely online projects have broadly focused on STEM (Science, technology, engineering, and mathematics) topics^32^. Furthermore, little is known about the demographic backgrounds and motivations of people taking part in citizen science projects concerning vector arthropods, which relate to both public health and biodiversity monitoring. Yet these projects can be particularly informative in studies of participant structure and motivation, because engagement may be influenced by personal concern in the immediate living environment through vectors (illness or fear of it, reduction of quality of life through nuisance). This personal interference possibly plays only a minor role in purely biodiversity monitoring initiatives, where, at least in large-scale, unstructured programs, the concern may be rather about the ecological crisis in general.

Calls for projects that engage the public in monitoring and mitigation efforts related to climate change have increased in recent years^33–35^, with significant attention given to research focusing on invasive species, including monitoring mosquito population variety and spread^36,37^, due to their threat to public health. For the latter, inclusivity is a specifically pressing issue as disadvantaged groups already suffer from health inequalities and could benefit from citizen science and community science approaches^38^. However, to the knowledge of the authors, only three studies exist that have investigated the motivations and/or background of participants of mosquito-related projects^37,39,40^. None of these were able to make comparisons in the motivation and demographics of participants with regard to different ways of involvement because the corresponding projects offered only one participation option by default, e.g. operating and taking care of a mosquito trap.

Therefore, this study aimed to gain insights into the demographics and motivations of volunteers participating in two citizen science projects that focus on similar goals, namely mosquito monitoring, but deploy different approaches: the digital Mosquito Alert (MA) and the analogue Mückenatlas (MS). Through a survey among the participants of each project, we compared (i) the demographic background, (ii) the motivations to take part, and (iii) the sentiments expressed when stating reasons to take part, in order to better understand who participates and why people participate. Due to differences in the projects’ participatory and communication designs, we expected that each program would attract different segments of the population despite pursuing similar goals. For the same reasons, we hypothesized that participants would exhibit varying motivations and sentiments toward their involvement in each of these projects. Diversity in citizen science projects is critical as its absence could have far-reaching scientific implications^15^. Therefore, our study aims to shed light on the impact of project designs on engaging different demographic groups in large-scale, unstructured citizen science programs. Specifically, our findings aim to identify the different sentiments that drive participants, enabling us to provide insights to other projects that aim to enhance the inclusivity and sustainability of their initiatives by responding to participant needs.

## Theoretical framework

Although most studies focusing on motivation in citizen science do not explicitly state an underlying theory^41^, a review of the literature shows that multiple frameworks, ranging from Baston, Ahmad, and Tsang’s community involvement^42^, Ajzen’s theory of planned behavior^41^ to the most commonly used model of Calary’s and Snyder’s Volunteer Functions Inventory (VFI) have been previously called upon^43^. In order to process motivational variations beyond exclusive categories, and avoid the exclusion of motivational goals, recent studies by Palacin et al.^44^ and Levontin et al.^43^ have made use of Schwartz’s Theory of Basic Human Values^45^.

The current study built upon this established groundwork and considered the Theory of Basic Human Values to classify open statements on personal motivations reported by respondents. This theory classifies motivations into four higher-order groups, constituting two bipolar dimensions. First, universalism or motivation that transcends self-oriented interests is contrasted with motivations to enhance selfish interests. Second, motivations for change are contrasted with motivations of conservation, i.e. the desire to keep things as they are. This framework has been previously used to develop motivational concepts, but does not adequately encompass the subject of goal orientation within its values. Therefore, we take into consideration regulatory focus^46^ to help us interpret citizen scientist sentiment, which as a theory of self-regulation differentiates individuals in terms of their goal-pursuit strategy. Focus is either more oriented to seek out growth (promotion focus) or avoiding loss (prevention focus). While a promotion orientation is characterized by striving to satisfy hopes, ideals, and the need to pursue positive outcomes, a prevention orientation prioritizes fulfilling obligations and avoiding failure to ensure safety and lack of negative results^47^. These desired end-states of decision-making are heavily based on how people respond to information according to their needs. Communication messages, e.g. from advertising slogans to the goals of citizen science projects, can be framed to match a regulatory focus. In effect, when considering a regulatory focus theory for citizen science, the triggering of either the prevention or the promotion focus would depend on how the project's messages are phrased. The digital and analogue mosquito-related programs of this study share the overall goal of mapping mosquitoes but convey different calls to action in their objectives and methods. This creates a unique opportunity to approximate potentially distinct sentiments underlying the motivations of their respective sets of participants and thus, to assess the applicability of the regulatory focus theory and expand on the existing frameworks of citizen scientists’ motivations.

## Methods

### The citizen science projects

Two of the most popular and successful mosquito monitoring projects in Europe are ‘Mosquito Alert,’ based in Spain, and ‘Mückenatlas’ based in Germany. Each uses a different appeal and approach for data collection. Mosquito Alert (MA) is a mostly digital project that tracks mosquito species by asking citizen scientists to take photographs of adult mosquitoes and submit them through a mobile phone application to an online platform. The app also collects volunteered metadata such as location and time of reporting and has the added functionality of letting participants report breeding sites and biting incidents. The objective of MA is to track the spread of vector mosquito species. The initial focus when the project was launched in 2014 was on following the spread of the invasive tiger mosquito, *Aedes albopictus* in Spain^48^ but it has since expanded to include other target species and a much wider global coverage. At the time of this study the target species were *Ae. aegypti*, *Ae. japonicus,* and *Ae. koreicus* as well as the common house mosquito, in addition to *Ae. albopictus*. The MA app is available in 18 languages, and it had been used to send reports from 175 countries, thereby contributing to the global monitoring of invasive mosquito species. The project is communicated via social platforms, a project website, and mass media. Participants receive direct feedback on their mobile phones from a group of expert entomologists who validate the photographs, and the expert-classified data is collectively shared in a public map from which it can be downloaded in various formats. Public health authorities in a number of countries use MA to detect the arrival of invasive vector species in new locations and activate health protocols and mosquito control measures once official confirmation is made^49^.

By contrast, ‘Mückenatlas’ (MS) maps the occurrence and spread of the complete mosquito fauna in Germany by increasing the number of locations and samples with the help of the public, although invasive species have been a focus of concern over the last years in Germany as well^50^. Another difference between MS and MA is that MS asks participants to submit physical samples through the postal service. (MA includes a module to facilitate the submission of physical specimens, but this is activated only in select countries and it is not at the heart of the project.) Physical mosquito samples are determined by experts only, and, in cryptic cases, genetically analyzed for identification to address various scientific questions. Briefly, people are asked to catch mosquitoes without damaging them, kill them by freezing, download and fill out a submission form with information on the date and location of the collection and send it (at their own expense) to the institutions involved (Leibniz Centre for Agricultural Landscape Research and Friedrich-Loeffler-Institut). The information from the mosquito catch is uploaded to a database for further analysis, and the participants are informed about the species submitted with a personal letter or e-mail. General communication about the project is mainly done through the dedicated website and mass media, which pick up press releases and conduct interviews with project leaders^51^. By the beginning of 2023, the project had received over 197.000 mosquitoes from more than 30.000 postal items. The mosquito data, however, is not immediately and generally made public. Upon submission of an invasive vector species from locations not yet considered colonized, the place of capture is checked by the MS working groups’ scientists or the informed responsible public authorities in due time in order to consider appropriate actions and control strategies^52^.

### The survey instrument

This study relies on surveys sent to participants between August and December 2020 for MS and August 2020 and September 2021 for MA, in both cases administered via KoboToolbox and offered in German or Spanish and Catalan^53^. In the case of MA, the link to the survey was delivered via the app to all registered participants in Spain and broadcast through the project’s Twitter account. In the case of MS, the link was embedded in the mail replying to submissions by the citizen scientists. The surveys contained questions concerning the participants’ demographic background, dwelling environment as in the physical characteristics of a person's living space, such as urban or rural, residential setting as in the inclusion of backyard, garden, or balcony where they reside, socioeconomic status, and relevant motivation categories. The variables on demographic background are based on a selection of the most relevant publications at the time of the design of the questionnaire and for which a connection between their characteristics and the motivation to participate in a citizen science project has already been assumed^20,23,54^. In general, age, gender, income, employment and education determine, for example, how much free time is available for citizen science activities or provide experience or skills that encourage participants to take part. These variables also play a role with regard to digitalization, especially age, making them particularly interesting for the comparison of the different project organization of MA and MS (digital and analogue). In addition, these variables make it possible to capture the diversity of the respondents and, accordingly, to estimate the inclusiveness of the projects and potential consequences on data quality. Living conditions, such as housing in a rural or urban area or having a garden/balcony, can influence attitudes and connections to nature and thus also be a factor in different motivations. These two variables are specifically relevant with regard to attitudes towards mosquitoes, i.e. whether participants see the animals only as a nuisance and pest or whether they are seen as an object of study in biodiversity research. Finally, living alone or with company such as with friends or family may decide also about the reasons to take part. With regard to mosquito-related citizen science projects, which are not only to be located in biodiversity but also in public health research, it could also be derived from this variable whether the monitoring of mosquitoes is possibly taken into one’s own hands for the protection of others.

Participants were asked to rate on a five-point Likert scale three motivation statements that were simplified from existing motivation categories^20,23,54^. We summarized aspects of understanding (learning, knowledge sharing) in the first, of values (support of environment, research and community) in the second and egoistic motives (personal enhancement), in the third statement. the participants were also asked to express in a few sentences why they participated in the respective citizen science project (see Supplementary Appendix S1 for all survey questions analyzed).Only with this open response question it was possible to learn about the project-specific motivations, including sentiments, and hence to apply our theoretical framework. In total, 410 responses were collected: 180 from MA and 230 from MS. Responses with missing fields were excluded during the assessment of each of the dimensions.

### Statistical analysis

For descriptive statistical analysis, a comparison of all demographic categorical variables between the projects was done using Chi-square tests to find out if the demographic profile of participants differed between the two groups, while for the closed response questions on motivation, a Likert scale rating^55^ of motivational factors within groups was performed using the Welch two-sample t-tests. The Welch t-test is a robust method of comparison for sample sizes that are not the same between the two groups and when the assumptions of equal variances are violated in independent samples like in our case. The surveys were designed in the local languages and the answers to the open-ended questions were translated into English via the open-access website deepl.com. During the blind assessments of the categorization, the authors, who are native speakers of German and Spanish, re-checked the answers in the original language for clarification in ambiguous cases. The answers were categorized into four distinct groups, based on four dimensions of motivators, each with two opposite extremes. The negative dimension of motivators relating to a tendency to focus on conservation and preservation, with the aim of maintaining the status quo, is contrasted by the positive dimension, which is associated with a willingness to embrace change and seek out new experiences. The other extremes were represented by self-oriented motivators, which include hedonistic motives and self-enhancement, and other-oriented motivators, which encompass values related to universalism and self-transcendence (Table 1). Initial coding was reaffirmed by an additional round of blind assessments done by two independent social scientists confirming initial allocations. The difference between the total number of responses assigned to each of the motivational categories was then analyzed using t-tests. Since our goal was to compare the average ratings of each motivational category between two different groups, rather than multiple raters within the same group, t-tests provided a suitable statistical approach. By using t-tests, we were able to evaluate whether there were significant differences in the average ratings, thus gaining insights into potential distinctions in perceived motivation levels based on the ratings assigned by participants in each project.

**Table 1:** Coding of open-response questions following our theoretical framework based on the theory of basic human values.

Motivation	Negative	Positive
Self-Oriented	Personal annoyance/fear of mosquitoes	Personal interest/curiosity
Other-Oriented	Public health concern/stopping the spread of diseases	Supporting research/scientific progress

Categories were built on the four higher-order value groups that guide behavior. Motivations were classified as either self- or other-oriented and associated with resistance (negative) or openness to change (positive).

The open statements were further analyzed by text mining and sentiment analysis. The AFINN lexicon which is a list of English terms manually rated for valence with an integer between − 5 (negative) and + 5 (positive) was used for sentiment analysis^56^. To complete the text processing, both sets of data were reshaped to a format where each row represents a single observation and each column represents a variable or feature of that observation to allow for text pre-processing. Stop words such as “the” “and” “a” and “is” were removed to better represent the underlying meaning of the texts, and the terms’ inverse document frequency was combined with their frequency to calculate their tf-idf (term frequency-inverse document frequency)^57^. The process produces a weight for each term in a document that reflects both the frequency of the term in the document and its importance in the corpus. In this way, it allows to measure how important and how unique a term or, a bigram—a sequence of two adjacent words—is.

For all tests, alpha (the threshold p-value for rejecting the null hypothesis) was set at 0.05. The final count of responses assessed varied between questions depending on the number of incomplete cases for each dimension. The fewest responses were given to the question about the employment status of the participants: 150 and 153 respondents from MS and MA, respectively. Data from the survey were analyzed using R version 4.0.3, with packages tidyr^58^, dplyr^59^, ggplot2^60^, tidytext^61^, stringr^62^, scales^63^ and vcd^64^.

### Ethical considerations

The methods used here were reviewed and approved by the Institutional Committee for Ethical Review of Projects (CIREP) at Universitat Pompeu Fabra prior to implementation (approval 161, 13 July 2020) and by the data protection officer of the Leibniz-Centre of Agricultural Landscape Research (ZALF), Germany. All research was performed in accordance with the General Data Protection Regulation (GDPR) on data protection and privacy in the EU and the European Economic Area, and informed consent was obtained from all survey respondents.

## Results

### Demographic variables

**Figure 1:**
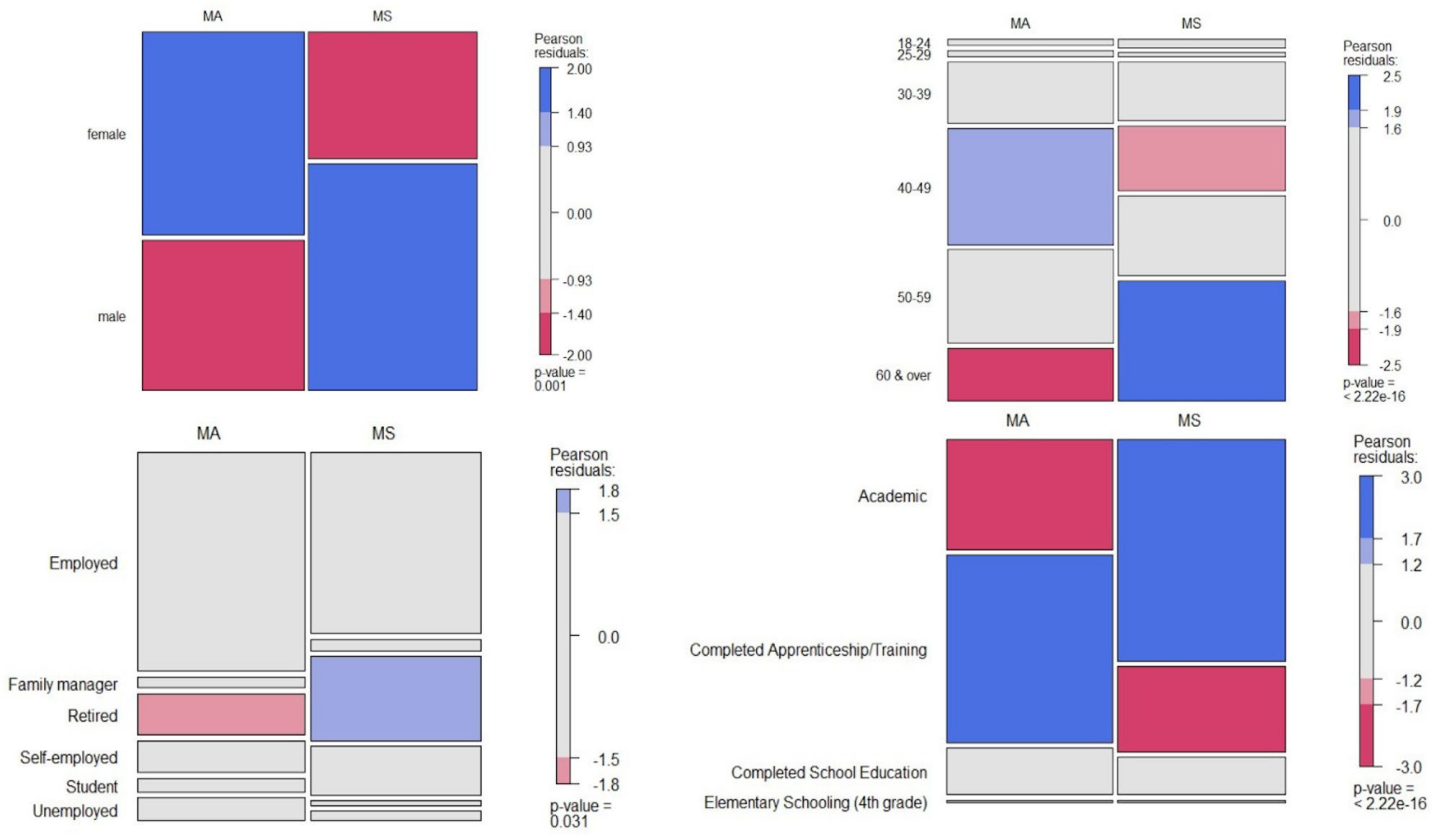
Mosaic plots based on contingency tables. Distributions of gender, age, employment status, and educational background among respondents of both project surveys. Statistics were performed by the Chi-square test of independence with Yate’s continuity correction. The number of responses assessed for this analysis for MS/MA were for 162/155, 160/159, 153/150 and 159/157 respectively.

Gender and age distribution between respondents were different. MA had significantly higher female participation, with 89 out of the 155 respondents (57.4%) being female compared to 58 out of 162 respondents (35.8%) of the MS survey (χ^2^ = 14.028, df = 1, *p*-value < 0.001). By contrast MS had a significantly larger pool of respondents aged 60 and older with 57 of the 160 respondents (36.6%) falling in that range compared to 25 out of 156 respondents (16%) for the MA survey (χ2 = 19.982, df = 5, *p*-value < 0.001). Furthermore, significant differences between MA and MS respondents could be found for employment (χ^2^ = 14.981, df = 5, *p*-value = 0.01) education (χ^2^ = 34.44, df = 3, *p*-value < 0.001) (Fig. 1), and income group (χ^2^ = 24.545, df = 10, *p*-value = 0.006) (Supplementary Fig. S1). The majority of respondents of both project surveys shared their living space, and, while participants of MS were equally distributed amongst various living environments, participants of MA were significantly less likely to live in a rural area (X-squared = 14.195, df = 3, *p*-value = 0.002) (Supplementary Fig. S1).

### Comparison of motivators

**Figure 2:**
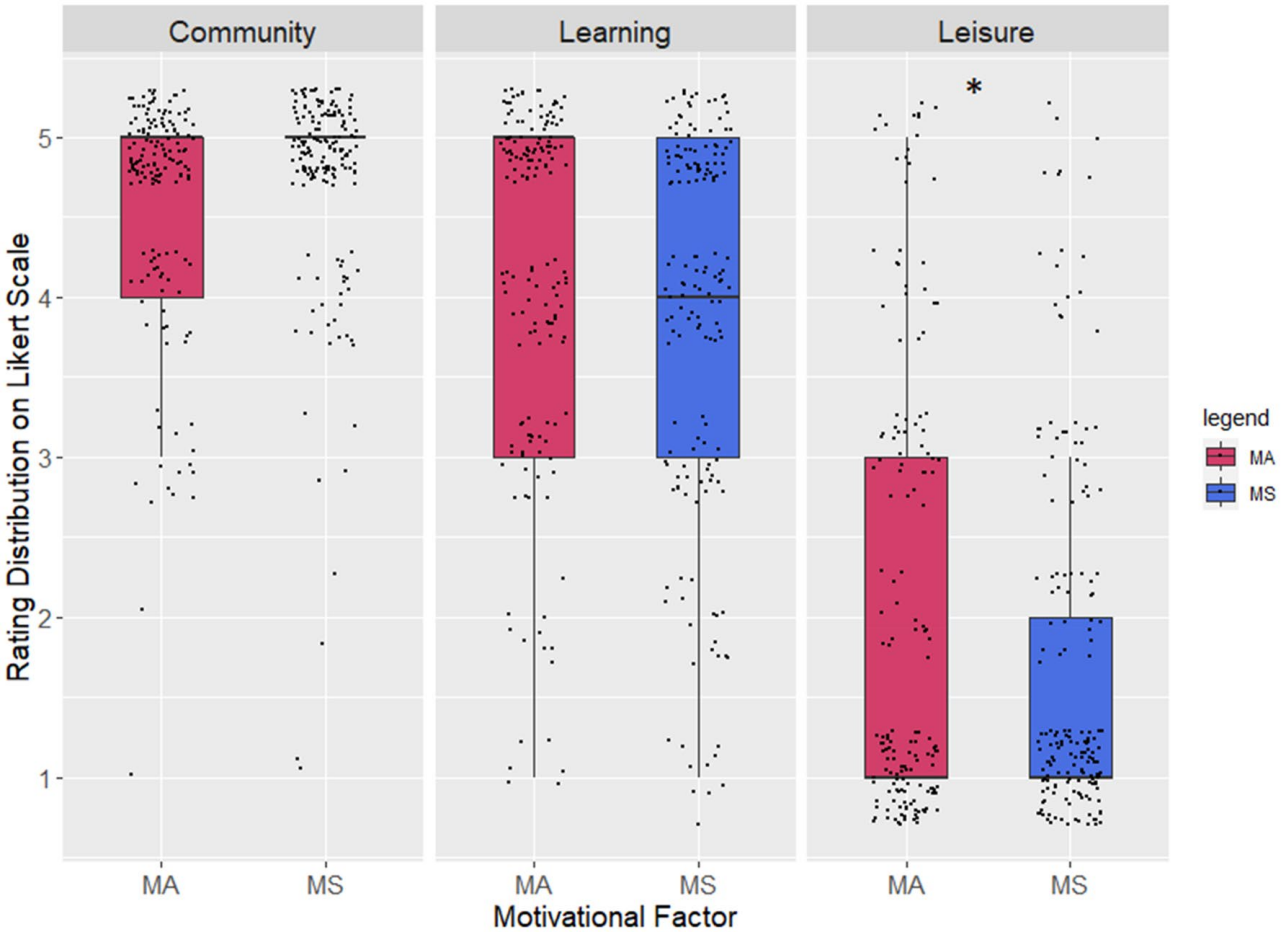
The distribution of ratings for motivational factors as reasons for participation. The participants’ assessment shows similar patterns of prioritization, with significant yet small differences in the rating of leisure. 170 and 159 responses were assessed from MS and MA respectively.

Out of the three given categories of motivations to participate, participants of both projects prioritized joining for the sake of the community (“I participate to support other people, research, and the environment”). The second most important reason to participate was to learn (“I participate in order to learn, to understand my environment and to share my own knowledge”), and lastly, for personal leisure (“I participate to improve myself, to solve a personal problem or to simply keep myself busy”) (Fig. 2). While the Welch Two Sample t-test showed no significant difference between participants of the two projects in their rating of involvement for the sake of the community and learning, leisure was rated as significantly more important for participation in MA (t (306.5) = 2.46, *p* = 0.014).

**Figure 3:**
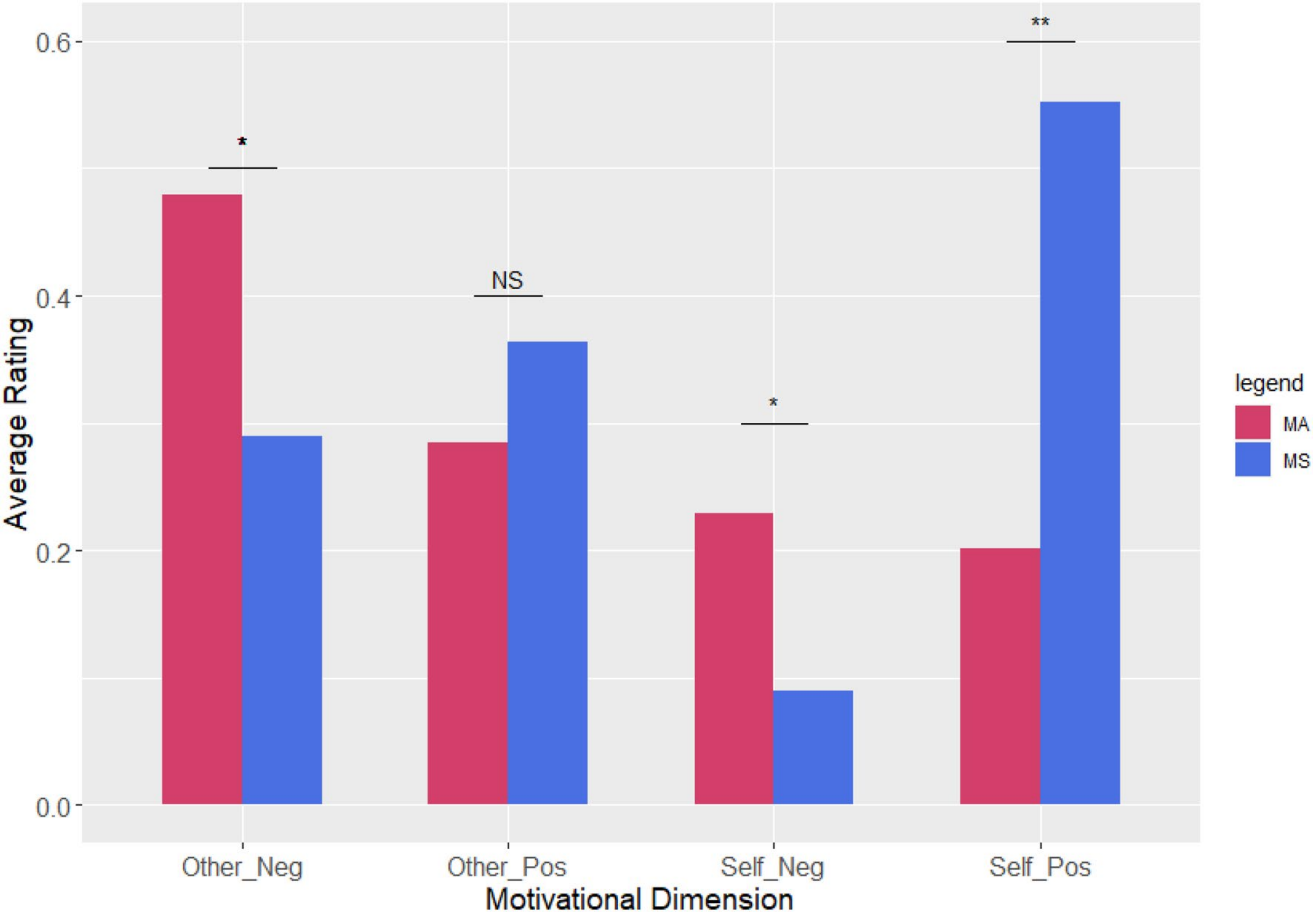
Average scores of the respondents’ coded answers. (*p < 0.1, **p < 0.05; 161 and 145 responses were assessed from MS and MA, respectively).

Analysis of the open-ended questions showed that MA participants were generally more motivated by the conservatory aspect of their participation while MS participants were more interested in the developmental aspect of their work (Fig. 3). The Welch two sample t-test showed slight differences between the projects regarding the influence of positive cues oriented other than their source of motivation to participate. Participation in MA was positively associated with both negative self-oriented motivators and negative other-oriented motivators (t (238.26) = 3.42, *p* < 0.001; Cohen’s d = 0.39 and t(291.26) = 3.56, *p* < 0.001; Cohen’s d = 0.40, respectively). Positive self-oriented motivators were significantly positively associated with participation in MS (t (330.65) = 7.12, *p* < 0.001; Cohen’s d = 0.78).

### Open response text-mining and sentiment analysis

**Figure 4:**
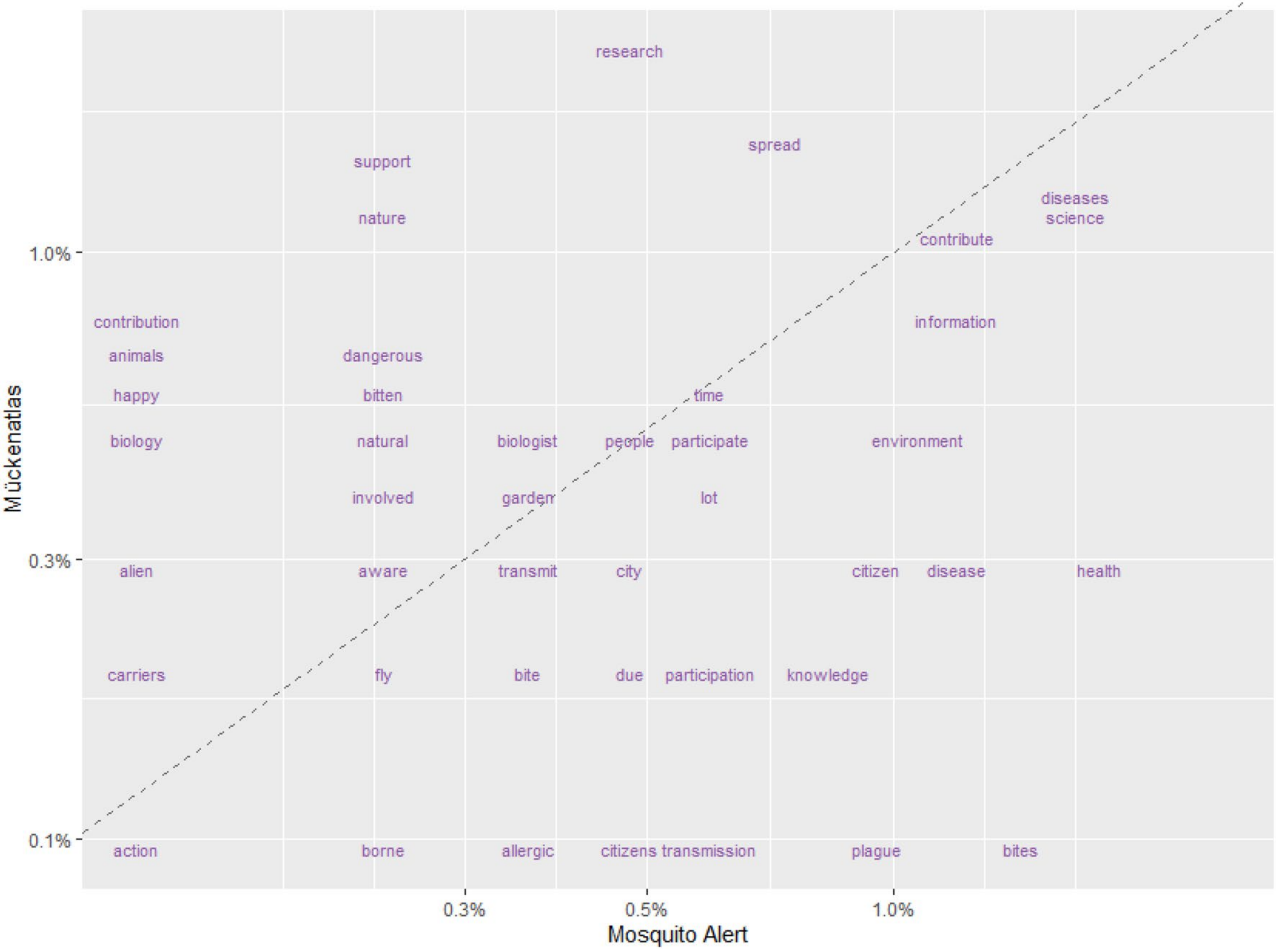
Comparison of word frequency used by participants of both projects. Words that are close to the line have similar frequencies in both sets of responses. The words further away are more unique.

Text mining analysis following Silge and Robinson^65^ revealed from the initial word count, that *control* was the most common word for MA, while also including terms such as *health*, *diseases*, and *bites*. As for MS, *research* was the most common word used by citizen scientists while also including terms such as *support*, *sciences*, and *nature*. Comparing the frequencies of words (Fig. 4) produced a clear cut between choices of words, with *plague*, *transmission*, *bite*, and *health* being more common for MA, and *research*, *support*, *nature*, and *contribution* being more common for MS. Pearson's correlation coefficient of terms between the two projects was 0.37.

**Figure 5:**
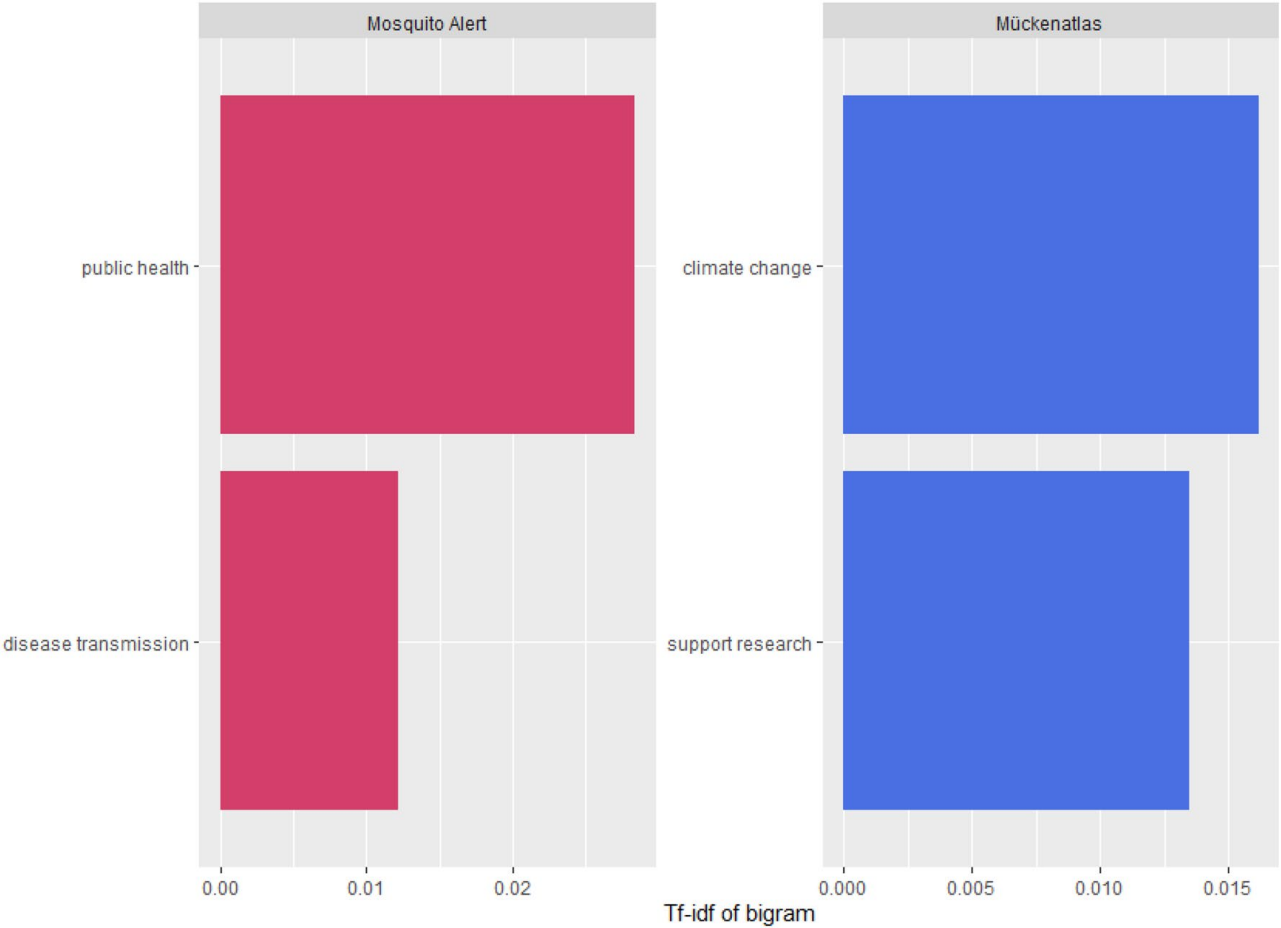
Bigrams with the highest tf-idf from each project. The most frequently occurring sequence of two adjacent words in open answer motivation responses of each project, weighted by their significance in the combined set of documents.

Further sentiment analysis (AFINN lexicon) provided evidence that MA participants are more likely to express negative sentiment and motivation to participate in order to counter a threat, while MS participants express a positive sentiment and are more likely to participate in order to push research, with the projects scoring with − 0.194 and 0.82, respectively. This variation in participant sentiment was further shown by the difference in their bigram’s TF-IDF (Fig. 5 and Supplementary Fig. S2).

## Discussion

Citizen science platforms covering a broad spectrum of scientific disciplines have become common, including projects supporting research in assessing and tackling the impacts of anthropogenic activities. While exploring the limits of what citizen science can achieve in terms of data collection, the various characteristics and motivational aspects of citizen scientists in relation to project design are simultaneously being researched^12^. Here we took the unique opportunity to compare demographic segments and motivations among participants of two projects that have the same research subject (mosquito monitoring) but two completely different methodologies. Determining what motivates participants and differentiates their engagement depending on the participatory approach, contributes to expanding the knowledge on how to sustain a project as well as on potential ways to address issues of exclusion.

As expected, we found significant differences in the demographic structure of both groups of respondents, in the form of gender, urbanity of the living environment, educational background, and income. More female participants responded to the MA survey, while more males answered the MS survey. In digital citizen science projects, female participants are more frequently motivated by health concerns than male ones^10^. As MA has emerged as an approach to help authorities monitor vector species, this could be a reason for the higher number of female participants, provided that the respondents’ gender distribution is representative of the population of participants in either project. Other health-related citizen science projects also report a higher proportion of women participating^66^, yet the data is still poor. Conversely, another reason for the higher proportion of men in MS could be that the project communication focuses more on the biodiversity than on the health relevance of mosquitoes and implements a collection process that closely aligns with the approach of natural scientists conducting field research. Men have been found to be overrepresented in many biodiversity programs^15^, also for other German insect-related projects^67^, but the picture is not uniform^68^.

Earlier studies stated that the majority of participants in citizen science projects are around 50 years old^15,69^. The MS respondents had a similar age structure with the largest segments of participants being between 60 and 50 years old respectively, in contrast to MA, where most respondents were in the age group between 40 and 49 years of age. The use of the MA app may pose a barrier for older individuals who lack the skills or access to use a smartphone or the internet, but it provides an easy and fast means of participation for younger segments of the population. The younger MA age structure could also be related to concerns about health, as described above, especially within families with younger children whose parents are worried.

More than half of the respondents to the MA survey reported not having an academic background. This differs from the literature according to which a majority of citizen scientists are higher educated^67^ and to which the results of the MS survey fit (> 57% with a university degree). The contrast between both projects in this regard may be reflected by different motivations and sentiment.

Our analyses of motivations by means of the predetermined statements to be rated and the open-ended response question partly supported our hypothesis that reasons for participation vary by project. Given response options with a Likert scale rating, the results of both projects’ respondents groups were similar. Supporting research and community was the main motivation for participation, followed by pursuing knowledge and leisure respectively. This agrees with what we know from the environmental volunteering literature and citizen science programs [e.g.,^20^^,^^54^]. Interestingly, Alender^70^ showed that the older the citizen scientists, the more the reasons to take part changed from self-oriented to self-transcendent reasons, which could explain the differences in the selection frequencies between MS and MA participants.

Categorizing the open response question (Table 1) indicated that participants in MA demonstrated a higher degree of motivation pertaining to external factors, especially ones aiming to maintain security, while MS participants were more likely to express motivations towards progress. Although both projects were successful in channeling different motivations that led to the participation and to the completion of the similar task of mosquito mapping, there were clear differences in sentiment. The MS participants were mostly driven by positive motivations, ranging from curiosity and willingness to learn to supporting scientific progress. MA participants were conversely driven by their antipathy of mosquitoes or their apprehension of a degrading environment and public health. This was corroborated by the sentiment analysis, which revealed higher frequencies of negatively charged words or bigrams, respectively. MA participants may be more interested in supporting mosquito control than in the science behind the project.

The divergent results can be interpreted with the differences in communication strategies and aims shared based on the initial intent of the researchers. Different project designs and communication strategies leading to similar end results in terms of participation frequency but appeals to pre-existing or influencing the motivations and sentiments of participants significantly. MA aims at surveilling mainly invasive mosquitoes^71^ and constitutes an early warning system that does inform research and health authorities to prevent the spread of these vectors. MA participants may have already been alarmed or became keen to react to goals that prevent negative emotions and conclusions. MS, on the other hand, is not negatively connoted by its name and aims at recording the entire mosquito fauna in Germany. The project (communication) is not directly associated with the health relevance of mosquitoes. In fact, public health is only addressed directly by the MS project leaders, when a first record or population of an exotic species has been discovered by the MS participants, which is then communicated to the public via the homepage or in a press release. Other than that, the topic of public health is mostly associated with MS in independent media reports on mosquito-related events^51^. By promoting positive aspects of science, the proportion of academics among the MS respondents is high and the participants’ curiosity is evident^52^. This distinct framing of projects might be similar to the division between the promotion-prevention focus in regulatory focus theory^46^, with MS emphasizing potential scientific gains, and MA the need to avoid public health risks helping attract distinct audiences. The participants’ aim to achieve similar end goals might be led on by distinct desired end states, one concerned with the prevention of negative consequences and the other with the promotion of positive ones.

## Conclusions

The demographic background, motivation, and sentiment of participants in projects with similar goals may vary significantly. We would like to emphasize here that—for citizen science projects—the MA App attracts an unusually high proportion of non-academic participants, as well as the hard-to-reach age group between 40 and 49 years. An easy-to-use app, which focuses less on the science and more on the control of invasive mosquito species, and that neither costs too much time nor money may attract a more diverse participant structure, while the time-consuming procedure of catching a mosquito sample might appeal more to a less occupied demographic group (e.g. seniors). While some may argue that using a simple app for citizen science misses the point of promoting scientific literacy among the public, it can be a convenient and accessible way to encourage participation from less privileged or time-constrained societal groups and trigger their interest in topics that they might have otherwise been forced to ignore. In fact, reducing the complexity of tasks required for participation has been shown to address the issue of time constraints, which is a significant barrier to participation for some groups. This can help to overcome underrepresentation of certain groups in citizen science, as reported in studies such as those by Domroese and Johnson^23^ and Nov et al.^19^. In addition to the aspects of inclusivity, a low diversity of the participants, as recorded with our selected variables, could lead to a non-realistic representation of the actual mosquito occurrence with consequences for the research results and the measures against invasive species^15^.

Furthermore, differing sentiments take root in a variety of motives that brought about the participation of citizens, highlighting the importance of investigating motivators beyond clear-cut categories. Participants of both projects were nearly identical in their prioritization of community and learning, but the in-depth text and sentiment analysis revealed clear contrasts between them. The findings do not necessarily call for prioritization of specific sentiments or changing of project-wide strategies for the projects investigated, as both are extremely successful in terms of the public’s responsiveness and scientific outcomes. Both key messages, in the case of MA on the issue of “public health” and in the case of the MS on the research gap “mosquito diversity”, seem to encourage many but different people in terms of their regulatory focus to record mosquitoes for science. The framing of the messages and objectives of MA rather seems to trigger prevention, whereas the participants of MS prioritize promotion. However, when planning or evaluating a citizen science project, it might be helpful to better understand how certain assertions of outreach activities relate to participants' intentions. Of particular interest would be to find out whether project communication appeals to pre-existing motivations leading to participation, or whether the messages adapt or even create motivators to take part. Further analysis of the potential split between the participants’ regulatory focus might be helpful to understand the relation between the alignment of project goals with participant motivators.

This study is limited by several factors; for example, a limited and possibly unrepresentative set of respondents. Since the survey also included other parts on recording and networking behavior (not evaluated here), the completion time was relatively long. In addition, e.g. for data protection reasons in the MS, it was not possible to contact all participants personally, but only to refer to the survey in the e-mails replying to mosquito submissions during the investigation period.

We partially excluded differences in the base population of Spain and Germany (see Supplementary Table S1), but this could not be done for all variables due to different data structures. Thus, it is possible that the differences between the type of employment and income group of the respondents are due to differences in the basic populations.

We also realize that the generalizability and evidence of our inferences based on two considered projects are limited. However, our focus was to compare two projects with similar scientific goals and the same research subject. Of course, there are other mosquito-associated projects around the world, the inclusion of which were not feasible for the lead authors due to the significant time and human resource investment required. Nevertheless, we believe that our results make an important contribution to the science of citizen science or at least provide thoughts for consideration and reflection. The innovative idea of this study was to compare a fully digital and fully analogue citizen science project with the same research subject to decouple information on participant structure and motivation from the influence of or the preference for the research subject itself, e.g., differing animal or plant species target(s). But not only does the project organization make a difference, the imbalance in project communication regarding biodiversity mapping and disease prevention also apparently has an equally strong influence on the motivation of citizen scientists. Overall, the experiences of the organizing scientists with the participants, i.e. the knowledge of who takes part and why, cannot be simply transferred from a digital to an analogue project and vice versa, even if the target taxa are the same and program objectives are quite similar.

## Supplementary Information

Supplementary Information.

## Data Availability

The datasets analyzed in the current study are not publicly available in order to protect the privacy of the citizen scientists' data, but are available from the corresponding author on reasonable request.
