# Supplementary material for: Demographic and motivational differences between participants in analog and digital citizen science projects for monitoring mosquitoes
Source: Sci Rep. 2023 Jul 31;13:12384. doi: 10.1038/s41598-023-38656-y (PMC10390545; doi:10.1038/s41598-023-38656-y)

**Supplementary Information**

**Demographic and Motivational Differences Between Participants in an Analog and a Digital Citizen Science Projects on Mosquito Monitoring**

Berj Dekramanjian^1^, Frederic Bartumeus^2,6,7^, Helge Kampen^3^, John Palmer^1,2^, Doreen Werner^4^, Nadja Pernat^4,5*^

1 Department of Social and Political Sciences, Pompeu Fabra Barcelona, Spain

2 Centre d’Estudis Avançats de Blanes (CEAB-CSIC), Blanes, Spain

3 Friedrich-Loeffler-Institute, Greifswald – Insel Riems, Germany

4 Leibniz-Centre of Agricultural Landscape Research, Müncheberg, Germany

5 Institute of Landscape Ecology, Animal Ecology Lab, University of Münster, Germany

6 Centre de Recerca Ecològica i Aplicaicons Forestals (CREAF), Barcelona, Spain

7 Institució Catalana de Recerca i Estudis Avançats (ICREA), Barcelona, Spain

**Supplementary Information**

**Table S1:** Gender, age group and living environment distribution of the total population of Germany and Spain. Data from: Eurostat (age, gender) and DEGURBA (cities = Densely populated areas (1), towns and suburbs: intermediate density areas (2), rural areas: thinly populated areas (2)).

|  | **Spain** | **Germany** |  |  |  |
| --- | --- | --- | --- | --- | --- |
| **Gender** |  |  |  |  |  |
| female | 24,171,413 | 42,128,512 |  |  |  |
| male | 23,227,282 | 41,026,519 | **𝜒2** | **df** | **p-value** |
|  | **Chi-square test** | | 0 | 1 | 1 |
| **Age group** |  |  |  | | |
| 18-24 | 3,348,241 | 6,161,121 |  |  |  |
| 26-29 | 2,531,860 | 4,912,939 |  |  |  |
| 30-39 | 5,410,496 | 9,744,336 |  |  |  |
| 40-49 | 7,085,224 | 9,005,560 |  |  |  |
| 50-59 | 6,376,132 | 12,126,697 |  |  |  |
| 60-69 | 4,862,813 | 9,455,306 |  |  |  |
| 70-79 | 3,549,880 | 6,514,328 |  |  |  |
| Over 80 | 2,531,706 | 5,097,510 | **𝜒2** | **df** | **p-value** |
|  | **Chi-square test** | | 72 | 64 | 0.2303 |
| **Living environment** |  |  |  |  |  |
| 1 | 25,403,040 | 30,003,919 |  |  |  |
| 2 | 15,381,263 | 34,263,020 |  |  |  |
| 3 | 6,241,905 | 18,752,274 | **𝜒2** | **df** | **p-value** |
|  | **Chi-square test** | | 6 | 4 | 0.1991 |

**Appendix S1:** Survey questions concerning the demographic background and the motivations of the citizen scientists. The survey questions were provided in German, Spanish and Catalan, respectively.

| **Survey question** | **Response (options)** |
| --- | --- |
| **Motivation** | |
| *With the next questions we want to better understand why people become citizen scientists.* *Please give us your reason(s) for participating.* | |
| Why did you participate in the Mückenatlas / Mosqito Alert? Please describe your personal reasons. | Open response question |
| *On a scale of 1 star (not applicable at all) to 5 stars (absolutely applicable): How much do these statements apply to you?* |  |
| I participate in order to learn, to understand my environment and to share my own knowledge. | Five star Likert Scale |
| I participate to support other people, research and the environment. | Five star Likert Scale |
| I participate to improve myself, to solve a per-sonal problem or to simply keeping myself busy. | Five star Likert Scale |
| **Demographic background** |  |
| *If you wish, you can answer demographic questions below so that in future we can design research projects with citizen participation in such a way that people from all socio-economic structures are addressed. Answering these questions is of course voluntary and just as anonymous; and you can always omit individual questions that you do not wish to answer, without your questionnaire losing its validity.* | |
| Would you define your living environment as rural or urban? | One of   - Rural - Rather rural - Urban - Rather urban - No answer |
| Do you have a backyard, garden, balcony or the like? | One of   - Yes - No - No answer |
| What is your gender? | One of   - Female - Male - Diverse - No answer |
| What is your age? | One of   - 18-24 - 26-29 - 30-39 - 40-49 - 50-59 - 60-69 - 70-79 - Over 80 |
| Do you live alone or in a community (e.g. family, partner, friends)? | One of   - Alone - With others - No answer |
| What is your highest level of education? | One of   - Completed primary school - I am in secondary school or have a completed school education - I am in training/apprenticeship or have completed a training/apprenticeship/master examination) - I am studying or have an academic title (bachelor, master/diploma, doctorate, professorship) - No answer |
| Would you please assign your monthly household income to one of the categories? | One of   - Less than 500 Euro - 500 to under 1000 Euro - 1000 to under 1500 Euro - 1500 to under 2000 Euro - 2000 to under 2500 Euro - 2500 to under 3000 Euro - 3000 to under 4000 Euro - 4000 to under 5000 Euro - 5000 to under 7500 Euro - 7500 to under 10000 Euro - 10000 Euro or more - No answer |
| What is your employment status? | One of   - Employed - Family manager - Retired - Self-employed - Student - Unemployed - No answer |

**Figure S1:** Mosaic plots based on contingency tables, showing distributions of a) living environment (𝜒2 = 15.234, df = 3, p-value = < 0.01) b) income (𝜒2 = 24.545, df = 10, p-value < 0.01), and c) cohabitation (𝜒2 = 4.7454, df = 1, p-value = 0.0293) among respondents of both project surveys (MA = Mosquito Alert, MS = Mückenatlas); statistics performed by Chi-square test of independence with Yate’s continuity correction.

a)


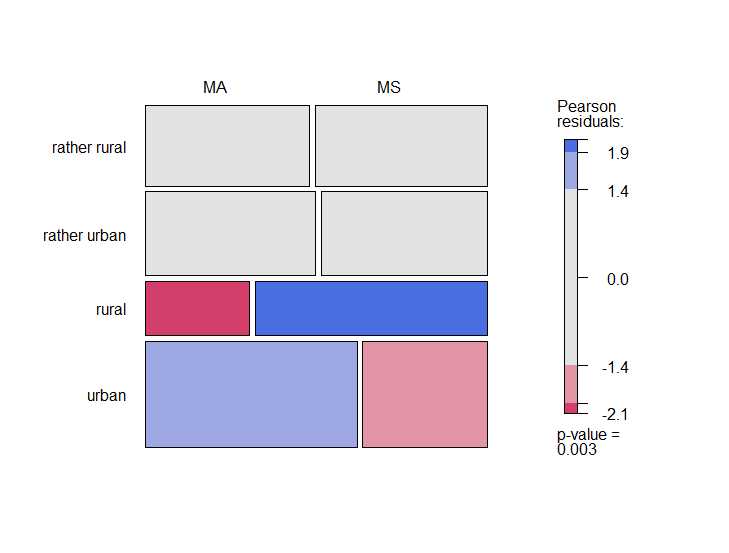


b)

c)
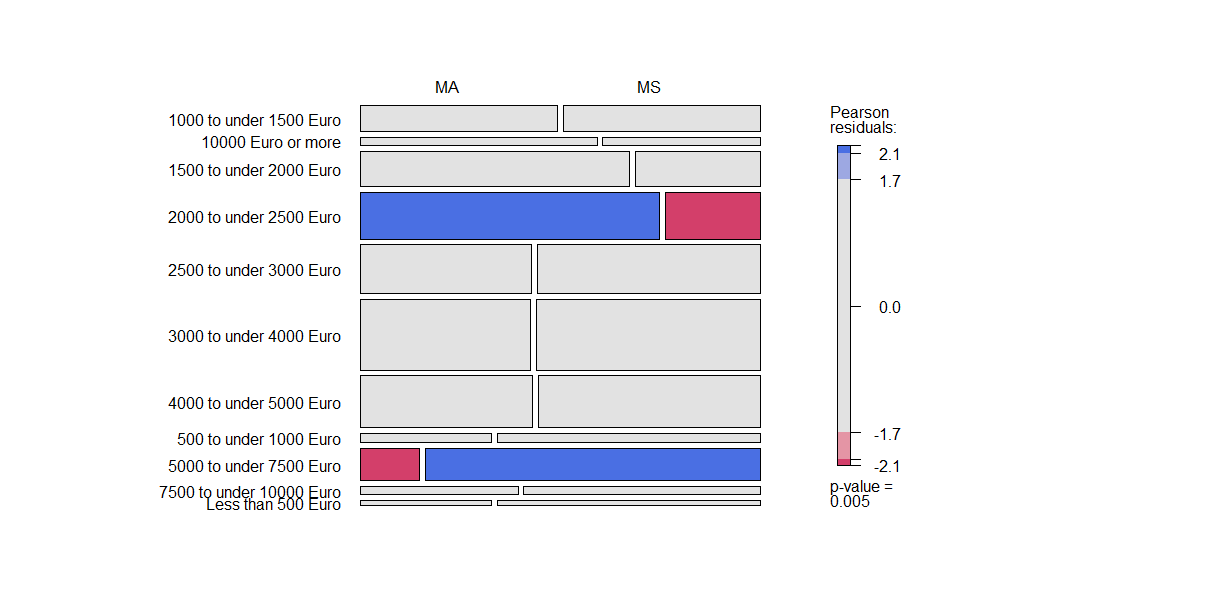


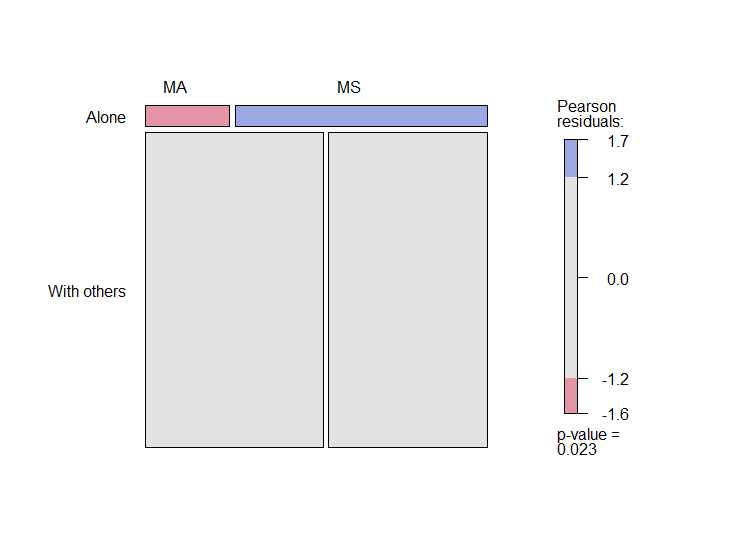


**Figure S2:** TF-idf bigrams


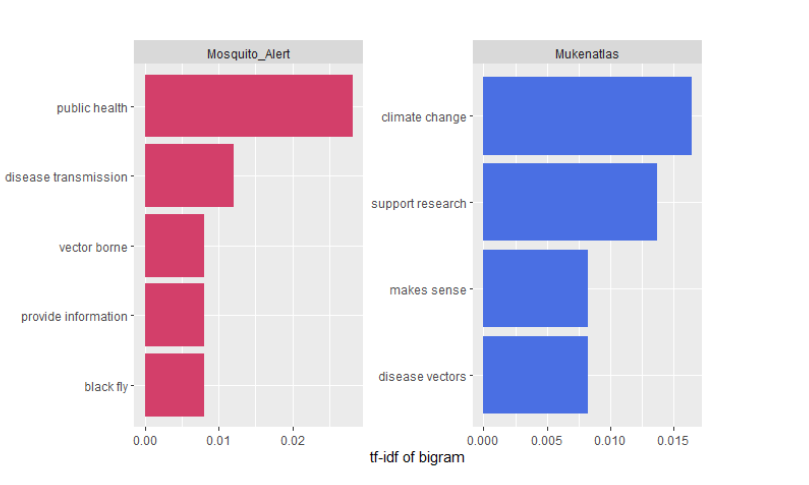

Supplement: Supplementary file 1 — Supplementary Information. [file 41598_2023_38656_MOESM1_ESM.docx]
